# Impact of Vascular Network Structure Heterogeneity on Retinal Tissue Oxygenation

**DOI:** 10.1007/s44007-025-00164-y

**Published:** 2025-06-05

**Authors:** Julia Arciero, Brendan C. Fry, Croix Gyurek, Amanda Albright, George Eckert, Gal Antman, Alice Verticchio, Brent Siesky, Alon Harris

**Affiliations:** 1 Department of Mathematical Sciences, Indiana University Indianapolis, 402 N. Blackford St, LD 270, Indianapolis, IN 46202 USA; 2 Department of Mathematics and Statistics, Metropolitan State University of Denver, Campus Box 38, P.O. Box 173362, Denver, CO 80217 USA; 3 Department of Biostatistics and Health Data Science, School of Medicine and Richard M. Fairbanks School of Public Health, Indiana University, 410 W. 10th St, Indianapolis, IN 46202 USA; 4 Department of Ophthalmology, Icahn School of Medicine at Mount Sinai Hospital, One Gustave L. Levy Place, Box 1183, New York, NY 10029 USA

**Keywords:** Mathematical model, Oxygenation, Retina, Vascular network, Heterogeneity, Glaucoma

## Abstract

A theoretical model of the human retina is simulated using two distinct vascular network geometries to predict the impact of heterogeneity in vascular network structure on retinal tissue oxygenation. Each vascular network is modeled as a combined heterogeneous representation of retinal arterioles and compartmental representation of capillaries, small venules, and large venules. A Green’s function method is used to model oxygen transport in the arterioles, and a Krogh cylinder model is used in the capillaries and venules. Identical input arterial blood saturation (0.92), arteriolar pressure drop (16 mmHg), and arteriolar diameters by vessel order (117, 73, 44, 32, and 22 µm) are assumed for both networks. The model shows that 12% of the arteriolar tissue in Branch 1 has a PO_2_ less than 25 mmHg, while only 1% of the arteriolar tissue in Branch 2 has a PO_2_ less than 25 mmHg. However, downstream of the capillaries, Branch 2 was predicted to exhibit lower tissue PO_2_ than Branch 1. The model also predicted increased oxygen extraction fraction as oxygen demand increased or capillary density decreased. Even with identical initial conditions for saturation, pressure drop, and diameter, variations in network geometry led to significantly different regions of low PO_2_, indicating a wide range of potential oxygenation outcomes for individual patients. This study therefore demonstrates that regional heterogeneity in vessel branching architecture may significantly impact oxygen saturation and ultimately retinal ganglion cell functionality, motivating the need for creating patient-specific vascular networks.

## Introduction

Impairments in retinal blood flow and oxygenation are involved in the progression of many ocular diseases, including glaucoma [1]. However, hemodynamics are not included as a standard of care in glaucoma management due to their complexity and void of large scale longitudinal data confirming vascular insults as primary drivers of glaucomatous disease. High individual variation in retinal structure and retinal vascularity has also inhibited comparisons of static biomarker values when attempting to estimate glaucoma risk. For example, an intraocular pressure (IOP) of a certain level in one individual may not determine risk in another individual with differing ocular structure or vascularity and/or resiliency to stress. Therefore, it has been difficult to develop conclusive evidence for the relative contributions of the multitude of vascular risk factors associated with glaucoma.

**Fig. 1:**
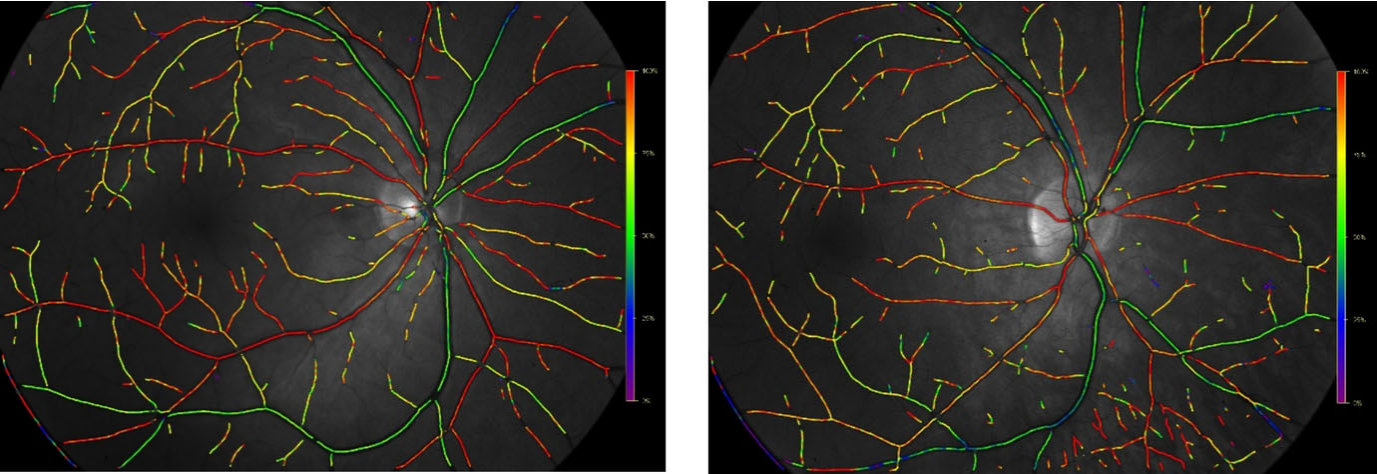
Oximetry maps obtained from two young, male, healthy individuals. Color map indicates oxygen saturation levels in arterioles and venules

Previous theoretical studies [2–4] have demonstrated that the heterogeneity of a vascular network leads to localized defects in tissue oxygenation that would not be apparent in the traditionally reported average values of tissue partial pressure of oxygen (PO_2_). As evidenced by oximetry maps (Fig. 1) and optical coherence tomography angiography (OCTA) images, the vascular network structure of the retina can differ among individuals regardless of their ocular health status. The oximetry images in Fig. 1 were obtained from two young, healthy males of non-Hispanic European descent. Despite the similarities in health and demographics between these two individuals, the positions and numbers of vascular branches downstream of the four primary retinal arteries were shown to differ between the individuals. We hypothesize that the resulting oxygenation of retinal tissue is impacted by vessel location and density, and that differences in these factors among individuals can impact retinal tissue health and function. Additional models and evidence are needed to substantiate the significance of these geometric differences on ocular health.

Previous mathematical studies of the retina have accounted for vascular network heterogeneity but do not include all aspects of the model presented in the current study. For example, in Causin et al. [5], the branching pattern for the simulated retinal vascular network uses a fractal structure with average fractal dimension of 1.7. While the value is obtained by averaging several factors, the network structure used in the study is not obtained directly from a physiological image. In addition, flow regulation mechanisms are not included in the model, and thus, the oxygen profiles in the vessels and throughout the retina are not influenced by the known vasoactive responses of arterioles. Liu et al. [6] obtained the positions and architecture of large arterioles from clinical fundus photography images (i.e., images obtained using a low-power microscope with camera capable of capturing images of the retina and superficial optic nerve head (ONH)). However, this approach was limited by camera resolution, leaving smaller arterioles in the image indistinguishable. Thus, smaller arterioles were represented in the model using asymmetric structured trees with self-similar binary bifurcations at each of the network outlets. Oxygen saturations throughout the network were predicted by the model, but flow regulation mechanisms were again not considered. The study presented here utilized physiological images of the retinal vascular network and incorporated flow regulation mechanisms to predict oxygenation of retinal blood and tissue.

The present study was designed to demonstrate the possible effects of patient-specific differences in vascular network structure on tissue oxygenation using a theoretical model of the human retina. The model was simulated using two different vascular network geometries. To make fair comparisons between the two networks without the potential for confounding effects of other patient-specific differences, the diameters of the same vessel branch orders were assumed to be equal, and the incoming flows and overall pressure drop across the arteriolar network were the same for both networks. The impact of vascular heterogeneity on blood flow and tissue oxygenation were assessed also in combination with other vascular factors, including varied levels of oxygen demand, flow regulation capacity, and capillary density.

## Methods

### Clinical Measures of Blood Saturation

As clinical motivation for this modeling study of the impact of vascular network structure on retinal oxygenation, assessment of bulk blood oxygen saturation was previously performed on a prospective data set from a cohort of 300 subjects, split evenly between glaucomatous and non-glaucomatous individuals [7, 8]. To measure retinal vessel hemoglobin oxygen saturation, clinical digital fundus photography was performed using mydriasis and flash imaging with a Topcon TRC-50DX Retinal Camera equipped with the Oxymap T1 retinal oximeter (Oxymap—Reykjavik, Iceland). Selected images were processed offline with Oxymap Analyzer (OA) (software version 2.5.2). Image data were filtered into discrete bandwidths, and images of vessels were recorded at oxygen-sensitive and -insensitive wavelengths. Arterio-venous difference was then calculated using the associated automated software. Optical densities (ODs) of vascular segments were determined using a computer algorithm to track the path of reflected light intensity along vessels. In graded hypoxia, the OD ratio (ODR = OD_sensitive_/OD_insensitive_) shows an inverse linear relationship to systemic oxygen saturation, with the arteriovenous saturation difference calculated as the difference in arteriolar saturation and venous saturation [9].

In addition to assessing possible differences in oxygen saturation based on glaucoma status, differences in oxygen saturation at different spatial locations within the retina were also assessed to explore whether spatial differences in oxygenation could be explained by the non-uniform spatial arrangement of blood vessels in different regions of the retina. The human retina can broadly be divided into four quadrants, based on physical location, with the ONH located at the center [10]. Clinical evidence has demonstrated that glaucomatous damage to the retinal nerve fiber layers (RNFL) progresses non-uniformly among the quadrants. Therefore, in order to assess whether there were corresponding blood oxygen differences in these different spatial locations, assessment of oxygen saturation in arterioles and venules of the retinal vasculature was performed in each of the four quadrants (see Fig. 3B): inferior nasal (IN), inferior temporal (IT), superior nasal (SN), and superior temporal (ST).

### Modeling Variability in Arteriolar Branching Structure

**Fig. 2:**
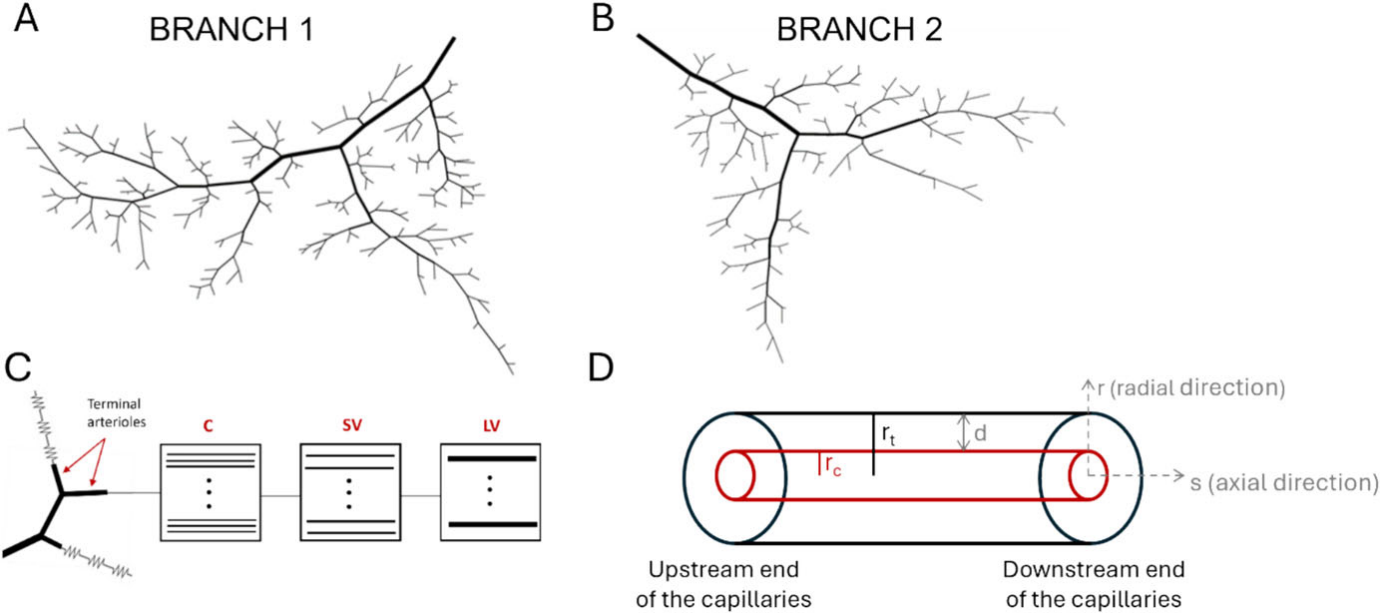
Schematic of vascular networks. **A**. Arteriolar network for Branch 1. **B**. Arteriolar network for Branch 2. **C**. Depiction of capillary (C), small venule (SV), and large venule (LV) compartments that are attached to each terminal arteriole. **D**. Depiction of Krogh cylinder tissue surrounding each capillary, where *r* is the radial direction, *s* is the axial direction along the vessel, *r*_*c*_ is the capillary radius, *r*_*t*_ is the tissue radius, and *d* is the tissue width

To investigate the hypothesis that differences in retinal oxygenation between glaucomatous and non-glaucomatous patients (or within the same patient between different ocular quadrants) can be explained by differences in vascular network geometry, this study used our previously developed theoretical hybrid model framework of the retina [4] to predict the impact of heterogeneous network geometry on retinal tissue blood flow and oxygenation. The original hybrid model includes a heterogeneous description of the arterioles and a series of compartments for the capillaries, small venules, and large venules. In this study, simulations were conducted using two different hybrid model networks, labeled “Branch 1” and “Branch 2” in Fig. 2A-2B. As described in detail in [2], these two network geometries reflect experimentally-observed branching patterns of vessels. The incoming artery for each branch feeds a non-uniform distribution of four to five orders of arterioles that differ in length and diameter before reaching a terminal arteriole. Identical input arterial blood saturation of 0.92 [11], arteriolar pressure drop of 16 mmHg [12], and arteriolar diameters by vessel order (117, 73, 44, 32, and 22 µm) [4, 13] were assumed for both branches. These assumptions were implemented to eliminate as many differences between the two networks as possible and to isolate the effects of branching structure.

Analogous to resistors in an electrical circuit, the compartments for capillaries, small venules, and large venules represent groups of identical parallel-arranged vessels that are considered to be fixed resistances (i.e., the diameters of the capillaries, small venules, and large venules do not change). These resistors/compartments are depicted in Fig. 2C and can be represented with the same notation used for resistors in an electrical circuit. To show more clearly the contents of these resistors, we also depicted the resistors as boxed compartments containing a set of parallel arranged vessels, where the dots indicate additional identical vessels. These capillary and venous compartments are connected in series to each terminal arteriole so that the entire retinal circulation is represented in the model. The fractional flows entering each series of downstream compartments of capillaries and venules are dictated by the flow emanating from the given terminal arteriole. This preserves the heterogeneity of the network downstream of the arterioles even without a heterogeneous network structure for the capillaries and venules. More specifically, flow in a single capillary (Q_C_) is based on an assumed capillary diameter of 6 µm, shear stress of 15 dyn/cm^2^, and viscosity of 9.05 cP. The number of capillaries in any given compartment downstream of a terminal arteriole is thus calculated as n_c,i_ = Q_TA,i_/Q_C_, where Q_TA,i_ is the flow through the i’th terminal arteriole.

### Modeling Hemodynamics, Flow Regulation, and Oxygen Transport

The hybrid model established in [4], which incorporates the elements of hemodynamics, flow regulation mechanisms, and oxygen transport mechanisms, is applied to each of the two heterogeneous vascular branches in this study. More specifically, each of the two heterogeneous vascular networks is represented as a directed graph, where each edge corresponds to a blood vessel of specified diameter and length. Flow through each vessel is driven by the pressure drop along the vessel and follows Poiseuille’s Law, $Q = \Delta {\text{P}}\frac{{{\pi D}^{4} }}{{128{\mu L}}}$, where *Q* is the flow rate in an individual vessel, ∆*P* is the pressure drop along the vessel, *D* is the vessel diameter, *L* is the length of the vessel, and *µ* is the apparent viscosity. Flow, pressure, hematocrit, and apparent viscosity are calculated for each segment using an iterative scheme [13]. First, based on a specified inflow (taken here as 40 mmHg) and outflow arteriolar pressure (taken here as 24 mmHg), conservation of flow is used to define a system of linear equations that is solved using successive over-relaxation for the pressures at each node. Flows are calculated assuming constant viscosity. Next, segment hematocrit and apparent viscosity values are calculated using the determined flows and pressures and assuming the conservation of red blood cell flow rate and plasma flow rate at each node. These schemes are alternately applied until convergence. At the output node of each terminal arteriole, the computed flow and pressure are used as the input flow and pressure to the corresponding downstream capillary compartment. Then, Poiseuille’s Law and conservation of mass are used to calculate the flow rates in the downstream capillary and venular compartments.

#### Modeling Flow Regulation

As described in detail in [3, 4], this model accounts for blood flow regulation by modeling the response of retinal arterioles to changes in pressure, shear stress, and metabolic demand [2, 3, 12, 14]. Briefly, a vessel wall mechanics model is implemented similar to [12, 14, 15], where the Law of Laplace dictates that the circumferential tension (*T*) within the vessel wall must balance the transmural pressure, which is the difference between the pressure inside the vessel (*P*) and the pressure outside the vessel (assumed to be the IOP in the eye).

1 $$ T = \frac{\Delta P \cdot D}{2} = \frac{{\left( {P - IOP} \right)D}}{2} $$

The total tension, *T*_*total*_, within the vessel wall is modeled as the sum of passive tension, *T*_*pass*_ (due to structural components and modeled by an exponential function of diameter) and active tension (due to contraction of smooth muscle cells and modeled by the product of a Gaussian function of diameter to represent the maximally active tension, T^max^_act_, and a sigmoidal function representing the activation, *A*, of vascular smooth muscle (VSM)), as shown in Eq. 2.

2 $$ T_{total} \left( {D,A} \right) = T_{pass} \left( D \right) + A\left( D \right)T_{act}^{max} \left( D \right) = C_{pass} e^{{C^{^{\prime}}_{pass} \left( {\frac{D}{{D_{0} }} - 1} \right)}} + AC_{act} e^{{ - \left( {\frac{{\frac{D}{{D_{0} }} - C_{act}^{^{\prime}} }}{{C_{act}^{^{\prime\prime}} }}} \right)^{2} }} $$

*D*_*0*_ represents the passive diameter of the vessel at a pressure of 40 mmHg. The level of smooth muscle activation (A_total_, Eq. 3) for a given vessel is modeled as a sigmoidal function of the input stimulus (denoted *S*_*tone*_, Eq. 4), which is assumed to be a function of the myogenic (pressure) response, the shear-dependent (flow) response, and the conducted metabolic (PO_2_) response.

3 $$ A_{total} \left( D \right) = \frac{1}{{1 + {\text{exp}}\left( { - S_{tone} \left( D \right)} \right)}} $$

4 $$ S_{tone} \left( D \right) = C_{myo} T_{total} \left( D \right) - C_{shear} \tau_{wall} \left( {D,Q} \right) - C_{meta} S_{meta} \left( {P_{O2} } \right) + C_{tone}^{^{\prime\prime}} $$

These responses cause a change in smooth muscle activation and, thus, vessel diameter. Model-predicted values for diameter and activation in each arteriole are calculated from the following system of differential equations for the *i*th arteriole:

5 $$ \frac{{dD_{i} }}{dt} = \frac{1}{{\tau_{d} }}\frac{{D_{c,i} }}{{T_{c,i} }}\left( {T_{i} - T_{total,i} \left( {D_{i} ,A_{i} } \right)} \right) $$

6 $$ \frac{{dA_{i} }}{dt} = \frac{1}{{\tau_{a} }}\left( {A_{total,i} \left( {D_{i} } \right) - A_{i} } \right) $$

where $\tau_{d}$ and $\tau_{a}$ are time constants, and *D*_*c*_ and *T*_*c*_ are the control (reference) state diameter and tension, respectively. At each timestep in the solving of Eqs. 5 and 6, the diameter of each arteriole changes in response to the flow regulation components of local pressure, shear stress, and PO_2_ (Eq. 4); the flows in each arteriole are then re-calculated according to Poiseuille’s Law, which then affect the flow regulation components, which then can cause an updated change in vessel diameter. This iterative solving process is repeated until a steady state is reached. Since blood flow is primarily regulated by the resistance vessels (i.e., arterioles), flow regulation in the capillaries and venular compartments is neglected. Parameter values for this model have been estimated and validated in previous modeling studies [2, 4, 12, 14, 16] and are given in Table 1.

**Table 1:** Parameter values for vessel wall mechanics (flow regulation) model

Description	Parameter	Value	Unit	Reference
VSM activation sensitivity	*C*_*myo*_	1.37/*D*_*0*_	cm/dyn	[16]
VSM shear stress sensitivity	*C*_*shear*_	0.0258	cm^2^/dyn	[12]
VSM metabolic sensitivity	*C*_*meta*_	1000	1/µM/cm	[3]
VSM constant	*C’’*_*tone*_	52–205		calculated
Passive tension strength	*C*_*pass*_	1.67·*D*_*0*_	dyn/cm	calculated
Passive tension sensitivity	*C’*_*pass*_	− 0.027·*D*_*0*_ + 12.52		[14]
Max active peak tension	*C*_*act*_	1.30·*D*_*0*_^1.48^	dyn/cm	[16]
Max active length dependence	*C’*_*act*_	− 0.00146·*D*_*0*_ + 1.13		[12]
Max active tension range	*C’’*_*act*_	− 0.00146·*D*_*0*_ + 0.308		[12]
Passive reference diameter	*D*_*0*_	29–151	µm	calculated
Time constant for diameter	$\tau_{d}$	1	s	[14]
Time constant for activation	$\tau_{a}$	20	s	[16]
Control state diameter	*D*_*c*_	22–117	µm	[17]
Control state tension	*T*_*c*_	13–188	dyn/cm	[17]

#### Modeling Oxygen Transport

Oxygen transport to tissue is modeled via the conservation of mass, which states that oxygen diffusion must equal oxygen consumption:

7 $$ D_{diff} \alpha \nabla^{2} P_{O2} = M_{0} \frac{{P_{O2} }}{{P_{0} + P_{O2} }} $$

where *D*_*diff*_ and *α* are the diffusivity and solubility of oxygen in the tissue, respectively, *P*_*O2*_ is the tissue partial pressure of oxygen, *M*_*0*_ is the tissue oxygen demand (i.e., maximum oxygen consumption rate), and *P*_*0*_ is the PO_2_ at which the consumption rate is half maximal (i.e., the Michaelis Menten half saturation constant). All parameter values are listed in Table 2.

**Table 2:** Parameter values for oxygen transport model

Description	Parameter	Value	Unit	Reference
Krogh diffusion coefficient	*D*_*diff*_* α*	6 × 10^–10^	cm^3^ O_2_/cm/s/mmHg	[18, 19]
Oxygen demand	*M*_*0*_	1—4	cm^3^ O_2_/100 cm^3^/min	varied
Michaelis Menten constant for O_2_ consumption	*P*_*0*_	10	mmHg	[20]
Tissue width of Krogh Cylinder	*d*	22–36	µm	calculated
Oxygen capacity of RBCs	$C_{0}$	0.5	cm^3^ O_2_/cm^3^	[21]
Inflow discharge hematocrit	$H_{D}$	0.4	-	[22]
Solubility of oxygen in blood	*α*_*b*_	3.1 × 10^–5^	cm^3^ O_2_/cm^3^/mmHg	[23]
Half saturation constant in Hill equation	*P*_*50*_	26	mmHg	[24]
Exponent in the Hill equation	*n*	2.7		[24]

In the arteriolar networks and tissue, this oxygen transport model is solved using a Green’s function method [13, 25–27] because it allows for the efficient prediction of oxygenation within a spatially heterogeneous network. Vessels are modeled as discrete oxygen sources, and the tissue regions are considered oxygen sinks. The resulting oxygen concentration at a tissue point is calculated by summing the oxygen fields (Green’s functions) produced by the surrounding blood vessels.

Oxygen transport in the capillary compartments is solved using a Krogh cylinder model, whereby each capillary within a compartment is represented as a Krogh cylinder surrounded by a sleeve of tissue. The capillary is assumed to deliver oxygen via diffusion to the surrounding tissue cylinder (see Fig. 2D). The governing equation for this process is the steady-state radial diffusion equation with consumption term:

8 $$ D_{diff} \alpha \left[ {\frac{1}{r}\frac{d}{dr}\left( {r\frac{{dP_{O2} \left( {s,r} \right)}}{dr}} \right)} \right] = M_{0} $$

where *r* is the radial distance within the tissue cylinder, *s* is the length along the capillary segment, and $M_{0}$ is the maximum oxygen consumption rate (assumed constant) in the capillaries. The width (*d*) of the Krogh tissue cylinder surrounding each capillary depends on the capillary density (*CD*), as follows:

9 $$ CD = \frac{{\mathop \sum \nolimits_{i} n_{C,i} L_{C,i} }}{{VOL_{A} + \mathop \sum \nolimits_{i} \left[ {n_{C,i} L_{C,i} \pi \left( {r_{C,i} + d} \right)^{2} + n_{SV,i} L_{SV,i} \pi r_{SV,i}^{2} + n_{LV,i} L_{LV,i} \pi r_{LV,i}^{2} } \right]}} $$

where the index *i* ranges over all vessel pathways (i.e., terminal arterioles, since a vessel pathway is defined as the path flow can take from the input of the network to the output (terminal arteriole) of a network), *n*_*i,j*_ represents the number of vessels in compartment *j* along pathway *i*, *L*_*i,j*_ represents the length of vessels in compartment *j* along pathway *i*, *VOL*_*A*_ is the total volume of the arteriolar network vessels and tissue (0.00115 cm^3^), and the summation in the denominator represents the total volume of the downstream vessel and tissue (i.e., the volume of the C, SV, and LV compartments). For a given capillary density, *d* is calculated using Eq. 9. In the reference case of *CD* = 500/mm^2^ [28], the tissue width surrounding each capillary is d = 22 µm. Since oxygen is exchanged primarily in the arterioles and capillaries, oxygen exchange in the venular compartments is neglected (*d* = 0).

In both the arterioles and downstream capillary and venular compartments, convective oxygen transport in the blood is also simulated. By the conservation of oxygen in each vessel segment,

10 $$ \frac{{df\left( {P_{b} } \right)}}{ds} = - q_{v} \left( s \right) $$

where

11 $$ f\left( {P_{b} } \right) = Q\left( {H_{D} C_{0} S\left( {P_{b} } \right) + \alpha_{b} P_{b} } \right) $$

is the convective O_2_ transport rate along a vessel segment, *Q* is the blood flow rate, *H*_*D*_ is the discharge hematocrit, *C*_*0*_ is the concentration of hemoglobin-bound oxygen in a fully-saturated red blood cell, *P*_*b*_ is the blood PO_2_, *α*_*b*_ is the solubility of oxygen in blood, *S*(*P*_*b*_) is the oxyhemoglobin saturation related to blood PO_2_ by a Hill equation $S\left( {P_{b} } \right) = \frac{{P_{b}^{n} }}{{P_{b}^{n} + P_{50}^{n} }}$, *s* is the distance along the segment, and *q*_*v*_*(s)* is the rate of diffusive O_2_ efflux per unit vessel length, as described in detail in [3, 4]. These oxygen transport parameter values are given in Table 2.

### Model Simulations

To assess the impact of vascular network structure on oxygenation, model simulations were run on the two networks under a range of conditions. PO_2_ was predicted in the arterioles and surrounding tissue and downstream of the capillaries under control (baseline) conditions as well as under conditions that may be observed in glaucoma patients such as increased tissue O_2_ demand, decreased capillary density, and impaired blood flow regulation.

As an additional way to compare oxygenation status of the different vascular networks under varying conditions, the oxygen extraction fraction (OEF) in the two networks was calculated, where OEF is defined in Eq. 12 to be the ratio of oxygen consumption to oxygen delivery:

12 $$ OEF = \frac{{\mathop \sum \nolimits_{i = 1}^{m} Q_{i} \cdot \left( {SaO_{2} - SvO_{2,i} } \right)}}{{Q_{total} \cdot SaO_{2} }} $$

where *m* is the number of terminal arterioles (and pathways), *Q*_*i*_ is the blood flow through the *i*th pathway, *SaO*_*2*_ is the incoming oxygen saturation to the arteriolar network, *SvO*_*2,i*_ is the oxygenation saturation at the downstream end of the capillaries along pathway *i*, and *Q*_*total*_ is the total flow into the arteriolar branch.

All simulations in this study are run using C code, and the Green’s function method was run on a parallel-processing GPU to decrease computation time. Repositories for the flow and Green’s function methods can be found at https://github.com/secomb/NetFlowV2 and https://github.com/secomb/GreensV4_GPU.

## Results

### Clinical Measures of Arterio-Venous Saturation

**Fig. 3:**
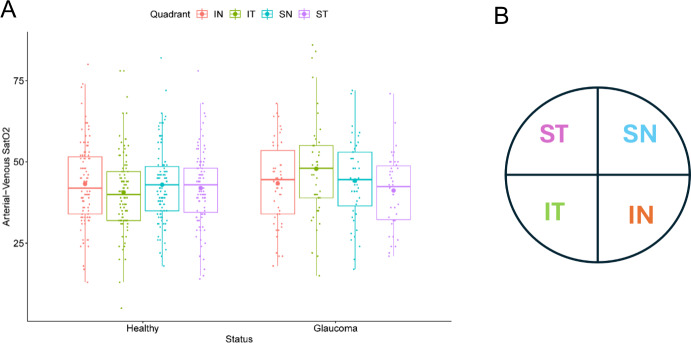
**A**. Summary of clinical measures of arterio-venous saturation difference between healthy and glaucoma patients by quadrant: inferior nasal (IN, red), inferior temporal (IT, green), superior nasal (SN, blue), and superior temporal (ST, purple). **B**. Schematic of quadrant positions of a right eye with the ONH located at the center of the quadrants

To examine spatial differences in oxygenation of the retina, we first looked at bulk oxygenation measures in the clinical cohort. The arterio-venous saturation differences among quadrants in healthy and early-stage glaucoma patients are depicted in Fig. 3. The arterio-venous saturation difference increases slightly in glaucoma patients, demonstrating lower venous saturation. Another result of interest is the predicted saturation differences by quadrant between healthy subjects and glaucoma patients. For example, the arterio-venous saturation difference in the inferior temporal quadrant (IT, green) was lower than the other quadrants in healthy patients but conversely was higher than the other quadrants in glaucoma patients.

### Geometric Variations in Retinal Microcirculation

**Fig. 4:**
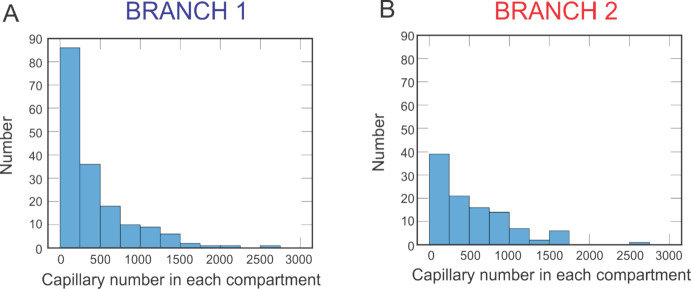
Histograms indicating the number of capillaries in the compartments downstream of each terminal arteriole. **A**. Number of capillaries in each capillary compartment in Branch 1. **B**. Number of capillaries in each capillary compartment in Branch 2

The spatial variation in bulk oxygenation measures in clinical data, such as the arterio-venous saturation difference provided in Fig. 3, motivates the need to further investigate the effects of geometric variations in the retinal microcirculation. To simulate such geometric variations in vascular structure within and between vascular networks, the theoretical hybrid model described in Sects. 2.2 and 2.3 [4] is used to predict oxygenation in retinal blood and tissue using two different hybrid model networks. As an initial analysis of the vascular structural differences between the two networks, the histograms in Fig. 4 describe the range in the number of capillaries in each compartment downstream of the terminal arterioles. The capillary number assigned to each compartment is a function of flow through the terminal arteriole, and thus the spatial heterogeneity of the arterioles is preserved through flow distribution downstream despite using compartments to model those vessels. Branch 1 is predicted to have 69,961 total capillaries compared to 56,844 in Branch 2. While Branch 1 has noticeably more capillaries than Branch 2, the distribution of capillaries per terminal arteriole is similar. In both branches, while the majority of pathways contain fewer than 500 capillaries (Branch 1 has a median number of 243 capillaries, and Branch 2 has a median number of 388 capillaries), both branches contain at least some pathways with over 2500 capillaries, demonstrating the wide variation in vascular geometry even within a single network.

### Impact of Vessel Heterogeneity on Tissue Oxygen Levels

**Fig. 5:**
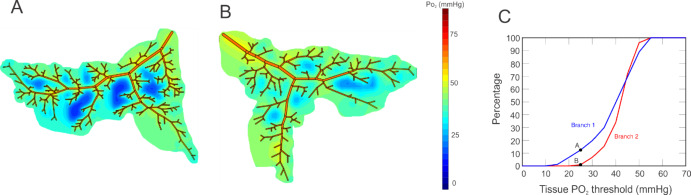
Comparing oxygenation of arteriolar tissue in two different branches that are assigned equal diameters at each vessel order to allow for fair comparison. **A**. Arteriolar tissue PO_2_ in Branch 1. **B**. Arteriolar tissue PO_2_ in Branch 2. **C**. The percentage of arteriolar tissue exhibiting a PO_2_ level below a specific threshold for Branch 1 (blue) and Branch 2 (red). Points A and B indicate that 12% of the arteriolar tissue in Branch 1 and 1% of the arteriolar tissue in Branch 2 has a PO_2_ less than 25 mmHg

Figure 5A and B are contour plots of the model-predicted levels of vessel and arteriolar tissue PO_2_ for Branch 1 and 2, respectively, for an oxygen demand of M_0_ = 3 cm^3^ O_2_/100 cm^3^/min. The color scale quantifies the PO_2_ in mmHg at different positions along the vessels or in the arteriolar tissue, with hotter colors (red, orange) indicating higher oxygen levels and cooler colors (green, blue) indicating lower oxygen levels. In Fig. 5C, the percentage of arteriolar tissue exhibiting a PO_2_ level less than a given value (PO_2_ threshold) is shown for Branch 1 (blue) and Branch 2 (red) for an oxygen demand of M_0_ = 3 cm^3^ O_2_/100 cm^3^/min. As indicated by points A and B, 12% of the arteriolar tissue in Branch 1 has a PO_2_ less than 25 mmHg, while only 1% of the arteriolar tissue in Branch 2 has a PO_2_ less than 25 mmHg.

**Fig. 6:**
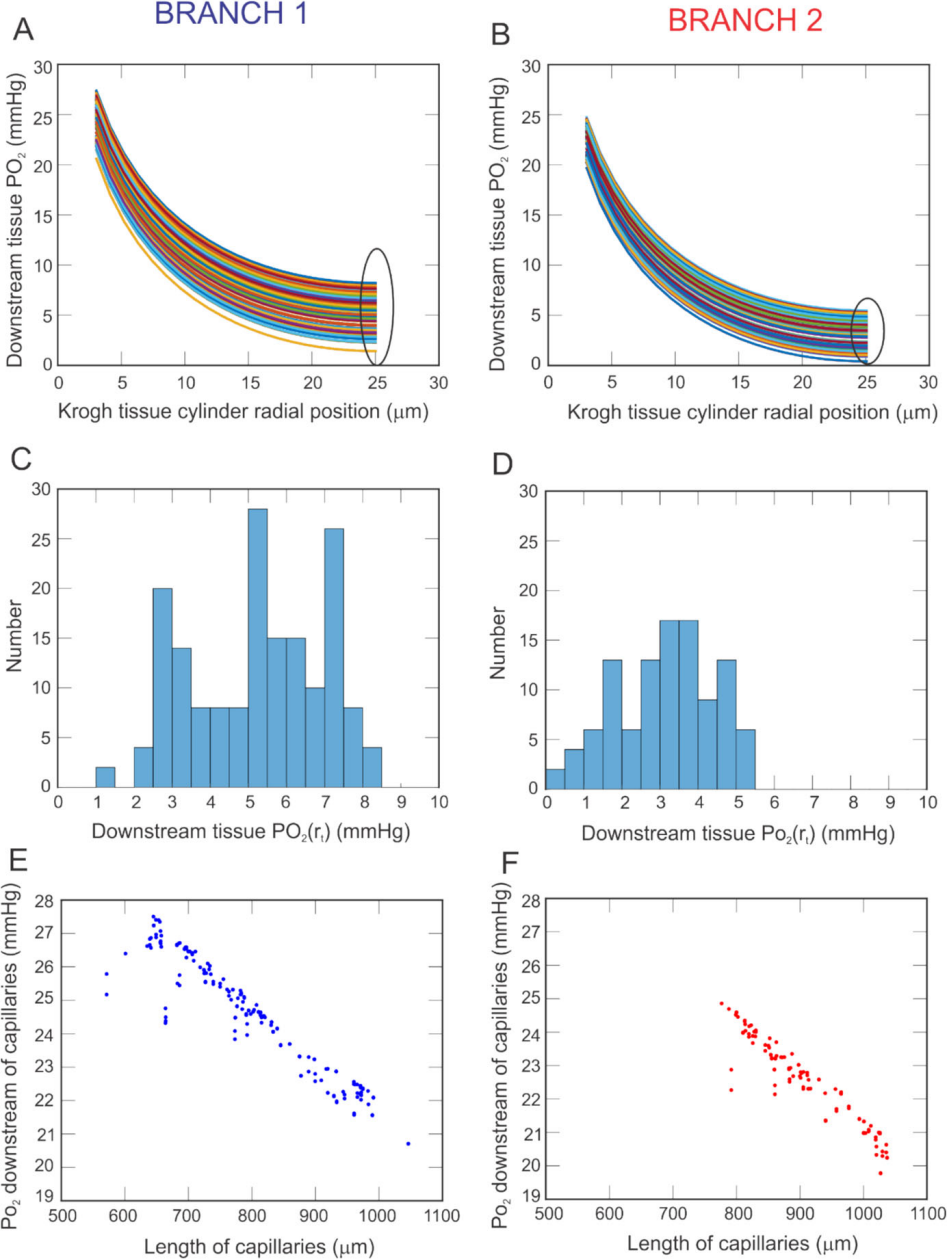
PO_2_ predicted in the Krogh tissue cylinder at the downstream end of the capillaries for each vascular pathway in Branch 1 (panel **A**) and Branch 2 (panel **B**). Histograms showing the spread in PO_2_ at the tissue edge (circled regions of panels A and B) are given for Branch 1 and 2 in panels **C** and **D**, respectively. Negative relationship between downstream PO_2_ value and capillary length is shown for Branch 1 (panel **E**, blue) and Branch 2 (panel **F**, red)

While Fig. 5 focuses on local arteriolar tissue effects of heterogeneity in vascular structure, Fig. 6 provides predictions of tissue oxygenation downstream of the capillaries (the site of primary oxygen exchange) for an oxygen demand of M_0_ = 3 cm^3^ O_2_/100 cm^3^/min along every vascular pathway. Panels A and B provide the values of PO_2_ in the Krogh tissue cylinder at the downstream end of the capillaries as a function of the radial direction within the cylinder, where r_c_ indicates the radial position of the edge of the capillary wall and r_t_ indicates the radial position of the outer edge of the tissue cylinder (positions labeled in Fig. 2D). Since a given capillary compartment is represented by a set of capillaries arranged in parallel that are all equivalent, every capillary within a given compartment has the same downstream PO_2_. As expected, PO_2_ levels drop from the edge of the vessel into the tissue in all pathways and in both branches. At the capillary wall, PO_2_ in Branch 1 ranges from 20–28 mmHg, depending on the pathway, and falls to 1–9 mmHg at the outer edge of the tissue cylinder. In Branch 2, the PO_2_ falls from 19–25 mmHg to 0–6 mmHg. The histograms in panels C and D show the predicted spread in PO_2_ at the edge of the tissue cylinder (i.e., the circled region in panels A and B, respectively) for each branch. Branch 1 exhibits generally higher predicted PO_2_ values, although there is a noticeable spread in downstream tissue PO_2_ in both branches. The relationship between capillary length and PO_2_ at the downstream end of the capillaries is depicted in Fig. 6E and F. Branch 2 generally has longer capillary lengths than Branch 1. The negative trend indicates that PO_2_ decreases with length of the capillary, both in Branch 1 and in Branch 2.

### Impact of Vascular Heterogeneity in Combination With Other Vascular Risk Factors on Tissue Oxygen Levels

**Fig. 7:**
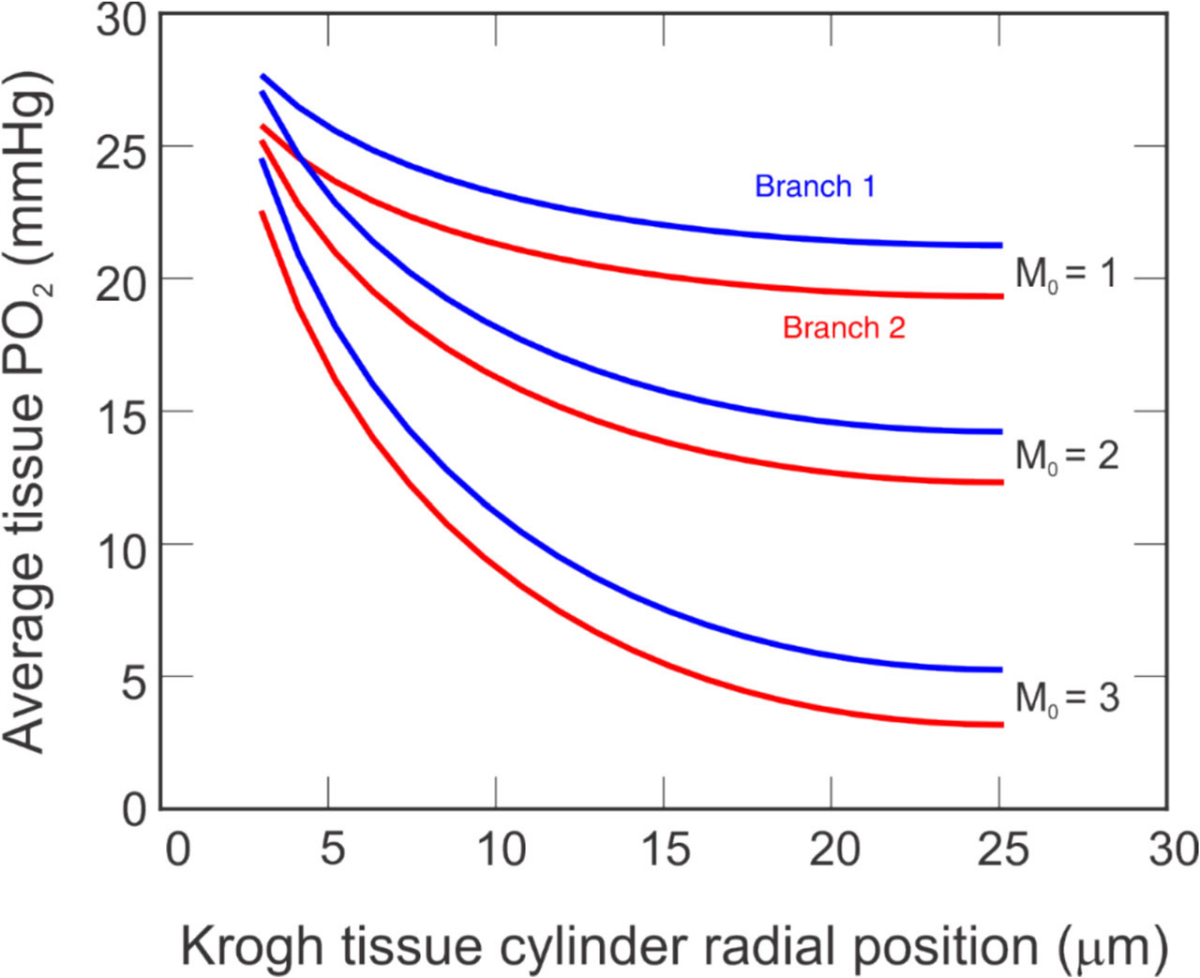
Mean tissue PO_2_ at the downstream end of the capillaries for Branch 1 (blue) and Branch 2 (red) for three oxygen demand levels: M_0_ = 1, 2, and 3 cm^3^ O_2_/100 cm^3^/min. Radial position represents the radial distance from the vessel edge through the tissue cylinder, as depicted in Fig. 2D

The tissue PO_2_ predicted in the radial direction of the Krogh cylinder depicted for each pathway in Fig. 6 can be averaged to give a general conclusion about tissue status for each branch. Figure 7 depicts these averages for Branch 1 (blue) and Branch 2 (red) for three levels of oxygen demand (M_0_ = 1, 2, 3 cm^3^ O_2_/100 cm^3^/min, labeled). Although the separation between the curves for Branch 1 and Branch 2 seems relatively constant over all radial positions, it is noted that there is a slight increase in the spread as the radius of the tissue cylinder is approached. In particular, the distance between the curves at the radius of the capillary and the radius of the tissue increases by 1.5%, 3%, and 4% for M_0_ = 1, 2, and 3 cm^3^ O_2_/100 cm^3^/min, respectively.

**Fig. 8:**
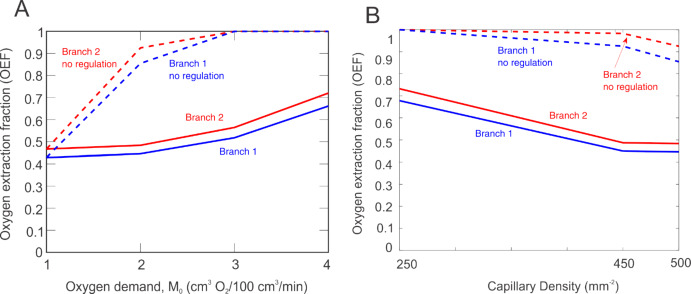
Oxygen extraction fraction (OEF) for Branch 1 (blue) and Branch 2 (red) with (solid) and without (dashed) regulation. **A**. Impact of oxygen demand (M_0_) on OEF with a baseline capillary density of 500 mm^−2^. **B**. Impact of capillary density on OEF for a moderate level of oxygen demand, M_0_ = 2 cm^3^ O_2_/100 cm^3^/min

Since glaucomatous patients have also been shown to exhibit other vascular impairments, including a reduced ability to regulate flow or a decreased capillary density, the model is used to predict oxygen extraction fraction (i.e., the ratio of oxygen consumption to delivery, OEF, as defined in Eq. 12) in the two different branches in the presence and absence of flow regulation mechanisms for variable oxygen demand (Fig. 8A) and for variable capillary density (Fig. 8B).

As depicted in both panels, the oxygen extraction fraction for Branch 2 (red) is consistently higher than Branch 1 (blue), regardless of oxygen demand level (M_0_) or if flow regulation mechanisms are functional (solid lines) or impaired (dashed lines). Although OEF is not directly comparable to the clinical arterio-venous saturation differences reported in Fig. 3, the model predictions indicate that variations in network structure impact retinal oxygenation and could thus potentially explain the observed differences in oxygenation between patient type or between different ocular quadrants of the same patient.

## Discussion

### Clinical Perspectives from Theoretical Predictions

The retina is the most metabolically active tissue in the human body, requiring a constant supply of oxygen to meet cellular demands. Previously, lower biomarkers of retinal blood flow and metabolism have been linked to ophthalmic disease including glaucoma [1]. Translation of hemodynamic insult in ophthalmic disease is currently impaired by a lack of specificity in outcomes tying specific tissue ischemia and hypoxia to future structural and functional degradation.

The mathematical model used in this study demonstrates the impact of heterogeneity of vascular network structure on the oxygenation of retinal tissue. The simulated vascular networks differed in the overall branching structure as well as number of vessels, as would be expected in individuals based on observations from retinal oximetry images (e.g., Fig. 1). Given identical initial conditions for incoming arterial saturation, pressure drop across the vascular network, and diameter for each branching order of arterioles, the model demonstrated that variations in network geometry can lead to substantially different regions of low PO_2_ in both arteriolar tissue (Fig. 5) and in the tissue at the downstream end of the capillaries (Fig. 6), indicating a wide range of possible oxygenation outcomes for individual patients. The differences that were observed between these two sample branches suggest that obtaining a patient-specific vascular map is critical in predicting and assessing potential risk of hypoxia or other vascular insult among different individuals. These observations may help explain why individuals with similar risk profiles, including those based upon biomarkers taken from global averages reported by standard modality software, have significantly different clinical outcomes.

This conclusion was further supported by clinical measurements that indicated a variation in predicted arterio-venous saturation differences not just between healthy and glaucoma patients but also within different quadrants of patients with the same disease status (Fig. 3). In other words, spatial locations and variations in network structure impact oxygenation within the retina, indicating that the observed differences in oxygenation both between healthy and glaucomatous patients and among the different quadrants of the same patient could potentially be explained by differences in vascular network structure in different patients or within different branches of the same patient. This study emphasizes the need for theoretical models to help reveal the impact of spatial location and variations in microvascular network structure on retinal oxygenation status. A higher level of specificity in digitally analyzed and modeled imagery may therefore improve clinical translation of hemodynamic biomarkers and better inform on the overall risk for glaucoma progression for each individual.

Interestingly, the simulated Branch 1 contained more arterioles (Fig. 2) and capillaries (Fig. 4) than the simulated Branch 2, yet the predicted arteriolar tissue PO_2_ was higher in Branch 2. At the downstream end of the capillaries, however, Branch 2 was predicted to exhibit lower tissue PO_2_ than Branch 1, again pointing to the impact of network-specific differences. Assessing the relationship between blood PO_2_ at the downstream end of the capillaries and capillary length demonstrated higher oxygen extraction in longer capillaries since transit time is larger (Fig. 6).

Model predictions also suggested that patients with certain vascular geometries may experience higher oxygen extraction than others and that this degree of oxygen extraction could be exacerbated at higher levels of oxygen demand, at lower values of capillary density, or if flow regulation mechanisms are impaired. For example, the oxygen extraction fraction at low M_0_ = 1 cm^3^ O_2_/100 cm^3^/min in Branch 2 is approximately the same as the oxygen extraction fraction at a higher M_0_ = 3 cm^3^ O_2_/100 cm^3^/min in Branch 1 (Fig. 8A). Similarly, the oxygen extraction fraction in Branch 2 for a capillary density of 500 mm^−2^ is similar to the oxygen extraction fraction in Branch 1 when capillary density is reduced by 10% (i.e., CD = 450 mm^−2^), as demonstrated in Fig. 8B. As shown in Fig. 8, the oxygen extraction fraction is always higher in Branch 2 than in Branch 1, but when regulation mechanisms are impaired, the oxygen extraction fraction approaches 1 for much smaller values of oxygen demand (M_0_) than if there were no impairment. Allowing functional regulation improves the situation in both branches but does not bring the curves any closer together. Thus, even when simulating optimal vascular conditions (such as increasing the capillary density or decreasing the oxygen demand), there are still differences in the predicted oxygen extraction fraction due just to the geometry of the vascular network.

When comparing average PO_2_ in the Krogh tissue cylinder at the downstream end of the capillaries (Fig. 7), the model predicted that the difference between the averages of the two branches increased slightly from the position at the radius of the capillary to the radius of the tissue. Additionally, this increase along the radial direction was larger for higher values of oxygen demand. These differences indicate the importance of accounting for individual variation in vascular anatomy of retinal networks when calculating a patient’s PO_2_ and making comparisons of oxygen status between different patients. Previous studies reporting global averages of patients and patient cohorts may not have revealed all significant relationships that are specific to regions of tissue affected by local tissue hypoxia. It is known that structure and hemodynamics of the different quadrants of the retina affect an individual’s vision in different areas, as assessed by mapping out their visual field [29] (i.e., a map of the total area in which objects can be seen in peripheral vision when the patient’s eyes are focused on a central point). Thus, isolating regions is likely to better inform on risk of disease based upon specific regional structure–function relationships most affected by diseases including glaucoma.

### Limitations

Although this study does not employ patient-specific vascular networks, this study provides a very important proof-of-concept based on two anatomically distinct physiologically based networks. The model demonstrates that the significant effects of vessel geometry should not be neglected when assessing potential for regions of impaired tissue oxygenation in the retina, especially in diagnostics of disease. Even with the same inputs (e.g., arteriolar diameters, flows, and saturations), the branching pattern led to different downstream effects. These results will be confirmed where possible over time with advancement of technology with clinically obtained values and studied in conjunction with longitudinal outcomes of disease onset and progression. Additionally, once images become available using ultrawide OCTA imagery of blood vessel geometry, patient-specific networks will be obtained and used as input into the mathematical model. Then, model-predicted oxygenation of retinal tissue will be validated against measures obtained from retinal tissue oximetry when available. Currently, the retinal photographic oximetry data obtained from our patient cohort only provides saturation measurements in large vessels, but not in microvessels or tissues.

Although not simulated here, the impact of vasodilators could also be tested using this model. We hypothesize that bulk treatment strategies such as vasodilators that would increase blood flow and dilate vessels would increase the average oxygenation in both networks but could exacerbate the gap in oxygenation between the two networks. Future studies might seek to understand the impact of network heterogeneity on differential therapeutic responses in patients with disease, especially for treatments that have vasoactive properties.

In the future, we plan to expand the model to include spatially dependent heterogeneous venous networks obtained from patient-specific images. However, it is expected that this additional complexity will have a much smaller effect on flow and oxygenation since the main site of resistance and regulation lies in the arterioles. In addition, this study did not include variations in IOP or incoming arterial pressure, in an attempt to isolate the effects of vasculature structure on flow and oxygenation. Future work will account for variable pressure, elevated IOP, and the potential for venous collapsibility.

### Concluding Remarks

This study demonstrates that regional heterogeneity in vessel branching architecture may significantly impact oxygen saturation and ultimately retinal ganglion cell health and functionality. Increasing specificity of vascular and metabolic assessment by sectoral analysis may help reveal previously hidden pockets of tissue hypoxia that are potentially creating cellular damage and vision loss seen in glaucoma and other ocular diseases.

## Data Availability

All data supporting the findings of this study are available from the authors upon reasonable request.
